# Paid care worker organizing in England: priorities and progress?

**DOI:** 10.3389/fsoc.2025.1548473

**Published:** 2025-03-14

**Authors:** Duncan Uist Fisher, Liam Foster

**Affiliations:** ^1^ESRC Centre for Care, The University of Sheffield, Sheffield, United Kingdom; ^2^Department of Sociological Studies, The University of Sheffield, Sheffield, United Kingdom

**Keywords:** adult social care, care worker organizing, neoliberalism, paid care work, trade unions

## Abstract

**Introduction:**

Despite its growth and ubiquity, paid adult social care (ASC) work in England persists as a site of very low pay, insecurity, and exploitation, where ‘decent work' remains elusive. Promoted by a neoliberal agenda focusing on competition and choice, social care provision has developed a quasi-market model. This involves local authorities assessing and commissioning predominantly independent sector providers to deliver care, which relies on outsourcing and contributes to workforce fragmentation. This atomisation, with thousands of providers and many workers employed to support people in their own homes, contrasts with the terrain of more established trade unionism and impedes organizing. Thus far in the English context, however, this phenomenon has received only limited attention in academic research. These challenges within the sector and limited organizing mean that it is important to understand priorities and progress in relation to ASC organizing.

**Methods:**

Thirty-five semi-structured interviews were conducted with key actors (organizers, administrators, founders) and paid direct care workers involved in organizing in the ASC context in England. Data were examined using thematic analysis.

**Results:**

The results identify four groupings where paid ASC workers and their representatives seek change: Pay and conditions; Systemic/structural change; Awareness-raising and being heard; and Environment and practices.

**Discussion:**

The discussion assesses the implications of these findings for ASC worker organizing and prospects for change. It contends that there remain significant barriers both to meaningful change in the situation of paid care workers, and to care worker organizing playing a greater or more prominent role in driving change. Concluding reflections consider what the issues identified in care worker organizing reveal about the relative status of care work and the circumstances of care workers, and paid care work's position in contemporary neoliberal capitalism.

## 1 Introduction

In England, adult social care (ASC) supports older adults, and adults of all ages with disabilities, to live their daily lives. Although closely connected to, and often overlapping with, healthcare, social care is distinct, and includes care and support with a range of activities, including household tasks, accessing services, and personal care (Baxter et al., 2020). Unlike the National Health Service (NHS), which has universal coverage and is free at the point of delivery, ASC is means-tested. Eligibility is judged not only on finances, but also on assessment of care needs, with these assessments undertaken by local authorities (Department of Health Social Care, 2024). Additionally, some ASC is funded through the NHS via continuing healthcare. Most care and support work is provided by friends and family through unpaid care, and there are estimated to be at least 5.8 million such unpaid carers in the UK (Carers UK, 2024, p. 4). The paid ASC workforce is comparatively small, but still hugely significant, amounting to 1.59 million workers (Skills for Care, 2024a, p. 15), the largest figure ever recorded (Skills for Care, 2024a, p. 29). ASC happens in different places: in people's own homes, in the community, and in care-specific settings, such as residential or nursing homes, supported or assisted accommodation, and daycare centers. As such, care work itself is spread across these locations and others, and the care setting plays an important role in shaping work content. Most people employed in ASC are “direct care” workers, and this is the group this article largely concerns: these are the core of ASC-specific, frontline workers who are either “entry-level” care workers or similar, or one step up in senior or supervisory roles (Skills for Care, 2024a, p. 12).

There are inequalities in relation to demographic representation in this workforce. For instance, 79% of the overall workforce are women (Skills for Care, 2024a, p. 73–75), underlining this employment's heavily gendered character (Bayliss and Gideon, 2020). Workers without British nationality are also significantly over-represented: the proportion of the workforce with British nationality is 75% compared with 90% of the population overall (Skills for Care, 2024a, p. 83). In terms of age, ASC has problems recruiting and retaining younger workers, who are under-represented, and there is concern at the number of workers nearing retirement age (Skills for Care, 2024a, p. 75–77).

As is common in many contexts globally, paid forms of care work in England are characterized by stubbornly poor pay and conditions, and a general undervaluation and lack of status (Glasby et al., 2021; International Labour Organization, 2018). These longstanding challenges have seen little by way of positive change or progress (Cominetti, 2023; Rubery et al., 2015). ASC is characterized by ongoing labor shortage and sustainability struggles (Skills for Care, 2024a), which make it particularly important to better understand the experiences of workers, and the challenges they face in their roles. Addressing these challenges is made harder by the fact that paid care workers lack a unified voice or influence through sectoral collective bargaining (Whitfield, 2022). Despite this, significant organizing and activist work toward improving ASC workers' situation is undertaken by these workers and organizations representing their interests (Johnson et al., 2021). In the English context, this phenomenon has received only limited attention in academic research. Therefore, this article focuses on what paid care workers and those who represent their interests seek to change regarding their work situation. This is done by exploring analytically, through the identification of themes, key issues that they organize around and their reasons for doing so. The empirical data consists of 35 interviews with paid care workers and key actors involved in organizing and union activity in ASC in England. The article considers what these identified issues reveal about the standing and quality of paid care work, and what they suggest about the prospects for meaningful change.

The novelty of our contribution is strengthened through our incorporation of paid care worker accounts, whose views on organizing are little understood (Whitfield, 2022). The article draws on literature on care and neoliberal capitalism to develop a novel analysis of this original data. It argues that strengthening our understanding of care (including advocating for its significance) as a form of resistance and opposition to capitalism in its present form is important for developing a holistic understanding of the challenges ASC workers encounter. In doing so it contributes to better understanding the needs and demands of ASC workers.

Initially, following this introduction, the article provides an overview of ASC structure and reform and their links with neoliberal capitalism. It connects the opposition paid care worker organizing represents to the notion of care being antithetical to contemporary capitalism (Lynch, 2022). It then notes the implications of these developments for the ASC workforce, before summarizing recent and current policy reform plans, and setting out how workers and those representing their interests respond to the current situation. The methods section follows, before the results, which identify four themes where paid ASC workers and their representatives seek change: pay and conditions; systemic/structural change; awareness-raising and being heard; and environment and practices. The discussion assesses the implications of these findings for ASC worker organizing and prospects for change, before concluding that there remain significant barriers both to meaningful change in the situation of paid care workers, and to care worker organizing playing a greater or more prominent role in driving change. That said, there are sources of optimism, and we highlight those and consider lessons, including on scalability and awareness of organizing.

### 1.1 Care and neoliberal capitalism

The circumstances of ASC workers, including many of the challenges they face, are situated within wider political and economic developments. This section outlines some of these developments and the specifics of how they have impacted ASC in a general sense. The outsourcing of much of ASC provision over recent decades has been key to a political economy focused on neoliberal policies to reduce state responsibility and increase the role of private markets and for-profit companies. This move is underpinned by the view that market forces, epitomized by competition and choice, will ultimately lead to improvements in cost-efficiency and quality (Corlet Walker et al., 2022). These key principles, now shaping the commissioning and delivery of care, link to wider moves to foreground neoliberal market ideology in the design and provision of foundational welfare services (Horton, 2022). The greater institutional certainty and rigid bureaucratic nature of the old state was deemed to be replaced by the “enabling state” (Gilbert, 2002). This represents a far more flexible entity that encouraged a new model of capitalism. In practice, the new dynamic structure has created greater uncertainty with its shift toward the financialization of welfare and individual responsibility (Berry, 2016; Foster and Heneghan, 2018). The broad motivation behind this move toward marketisation has been the idea that market forces will deliver improvements in cost-efficiency and quality, as well as providing consumers (i.e., service users) with a greater choice of providers. This is despite concerns, according to classical economic theories, that many of the conditions necessary for a market to produce optimal outcomes are not met by the health and social care sectors (Corlet Walker et al., 2022).

In ASC, these trends have led to a quasi-market (Le Grand, 1991) consisting of 18,500 (Skills for Care, 2024a, p. 16) provider organizations from which both local authorities and individuals purchase care (Curry and Oung, 2021). 79.1% of ASC providers are “independent” (Skills for Care, 2024a, p. 21), and of these 74% are private and 26% are charitable organizations (Skills for Care, 2024a, p. 22). This structuring sprang from legislation introduced by the Conservatives in the 1990s, characteristic of a neoliberal market economy, which redefined the role of local authorities as purchasers of services (Rummery, 2018). To take the example of homecare, in 1993 the private sector delivered 5% of it, but by 2012 this was 89% (Hudson, 2016). ASC work has become increasingly fragmented: in addition to the thousands of individual providers, there are local authority-level variations, and numerous care settings (e.g., residential, homecare, live-in care) and types of employer/models of employment. This results in the dispersal of workers (Murphy and O'Sullivan, 2021, p. 390).

This neoliberal paradigm is not just economic and political, but cultural. The liberalization of capital fostered a new “culture of capitalism” (Sennett, 2006). In Sennett's terms, this reorganized the institutional structure of firms and, consequently, they became more focused on the short-term to satisfy the needs of global capital. In ASC this has entailed dismantling institutional structures, reorienting roles, and overhauling structure. Furthermore, ASC has become one of the main areas where finance has come to shape public service delivery, especially since the early 2000s (Blakely and Quilter-Pinner, 2019). This has resulted in the creation of complex avenues for the extraction of shareholder returns, such as high payments for rent, and borrowing from companies in the same corporate group (Bayliss and Gideon, 2020). The role of marketization in the sector is also evident through the increased involvement of private equity firms, who now own several of the largest care home chains, not only in the UK, but also in Norway, Sweden and the USA, among other places (Harrington et al., 2017). This indicates the pervasiveness of challenges beyond the UK. Consequently, the increasingly elaborate extractive international financial architecture has transformed the relatively low risk and more straightforward process of providing social care services (Burns et al., 2016).

It has been argued that the role and value (economic and socially) of care and caring have been transformed by the neoliberal care agenda. The reorientation of care provision along neoliberal lines in contemporary capitalist society informs care scholars' wider concerns about the role of care and its opposition to, and incongruency with, the neoliberal capitalist social order (Lynch, 2022). Robinson (2013, p. 141), informed by and seeking to advance the influential care ethics literature, contends that “neo-liberalism is explicitly anti-care, since it views the giving and receiving of care a sign of failure, dependence or deviance.” Neoliberalism's “anti-care” (see also Lynch, 2022, p. 26–27), through the actions of states, institutions, and corporations, creates ever new circumstances where care becomes necessary, often in increasingly complex forms and grave settings. When this care is required, these actors do not adequately support those needs for care (and care workers), and they cede responsibility for it to individuals. The marketization of ASC is an example of the state ceding responsibility, and the lack of care manifests itself in decreased levels of state provision, the consequences for unpaid carers, and the treatment of paid direct care workers.

Prominent care scholar Tronto (2017) questions the inevitability of neoliberal dominance and proposes care as a grounding for alternative theorizations of society. She argues that neoliberalism's theory of care has three strands, which align with Robinson and the account of English ASC restructuring and marketization:

care for yourself by acting rationally and responsibly; if there are care needs that you cannot meet for yourself, then use market solutions; and, finally, if you cannot afford market solutions, or prefer to care on your own, then enlist family (and perhaps friends and charities) to meet your caring needs (Tronto, 2017, p. 30).

A key strand of Tronto's thinking is that care provides an alternative ontology to the rational actor basis for understanding human behavior. In particular, she highlights our mutual interdependence, and thus our “collective” (Tronto, 2017, p. 32) societal responsibilities. This notion of collective responsibility has clear relevance in the context of organizing, and to the efforts of workers and those who represent them to seek change in their collective circumstances.

Robinson, Tronto, and others, are aware of the injurious effects neoliberal capitalism's ordering of care imposes on particular people within society. This is evident not only in the varying degrees of exploitation of paid care workers, including across national borders, but in the ongoing unequal gendered, classed and racialised distributions of unpaid caregiving (Tronto, 2017, p. 38). Furthermore, care scholarship recognizes how these distributions and consequent inequalities are situated within historical and contemporary global hierarchies (Raghuram, 2012; Williams, 2018). These ideas allow for consideration of how the concerns of workers and those involved in organizing oppose present circumstances. Crucially, this scholarship also acts as a vehicle to interpreting wider concerns about paid care work's place within contemporary neoliberal capitalism, and its role in perpetuating social inequalities.

### 1.2 The implications of neoliberalism for ASC work and workers

This section more closely examines the consequences of neoliberal reforms for ASC work and, in particular, ASC workers. There are concerns around the implications of financialization and the abundance of for-profit care providers for quality of care and working conditions (Atkinson and Crozier, 2020; Corlet Walker et al., 2022), as well as ASC's economic and operational stability. Price constraints create pressures on margins, often leading to competition between shareholder profits and employee wages. Dromey and Hochlaf (2018) and Eurofound (2020) note strong evidence that private providers tend to have lower levels of staffing, higher staff turnover, lower rates of pay and lower levels of training. Furthermore, cost cutting measures by employers to maximize profit have often resulted in greater precarity and casualisation (Taylor et al., 2021), through the use of flexible and short-term contracts. This new employment landscape threatens job security, particularly in long-term employment in one firm or industry. These trends indicates that “labour is losing this fight with shareholders” (Corlet Walker et al., 2022, p. 301). Scholars have strongly criticized the state for its direct role in outsourcing failing to uphold ASC workers employment standards, and for the level of public funding allocated to ASC (Hayes, 2017). Hayes uses the term “institutional humiliation” to theorize these workers' employment degradations. There are concerns about the use of predatory financial practices resulting from the financialization of ASC, and its implications for working conditions and standards of provision (Corlet Walker et al., 2021). This also raises questions about the direction of government policy driving these trajectories, and levels of public funding for adult social care, regardless of forms of provision.

Research highlights the link between the quantity and quality of the workforce and the quality of ASC provision (Eaton, 2000). For instance, private providers are characterized by reduced staff time per resident, compared with the non-profit element (Eurofound, 2020). In theory increased competition between ASC providers could translate into pressures to innovate, enhance care quality, or devise cost-efficiency savings. However, modest opportunities for economies of scale in ASC delivery combined with the limited scope for labor productivity gains in the workforce, and low wages in the sector, limit providers' capacity to achieve cost-efficiencies without compromising care quality (Corlet Walker et al., 2021).

These trends, including how they undermine workforce sustainability and drive high turnover, have clear implications for paid care worker organizing and the areas of focus (Whitfield, 2022). Although the sector has been classed as low-paid by the Low Pay Commission since 1988 (Hemmings et al., 2024, p. 10), it is evident that the privatization of the sector and the principles associated with this has led to these remaining low. The very low basic rates of hourly pay are compounded by a range of other elements where these workers are poorly compensated. These include ongoing problems with payment for homecare workers' travel time between care visits (Cominetti, 2023, p. 25–30) and issues over payments for breaks and sleep-in work [Migration Advisory Committee (MAC), 2022]. There are examples of workers incurring other work-related expenses, such as insurance, parking, or fuel (Hemmings et al., 2022, p. 46). There is a general lack of incentive available to ASC workers: pay increases for promotion or length of service are often meager, and with the former, at times non-existent (Rubery et al., 2015, p. 765). ASC workers are short-changed on sick pay, with the legal minimal statutory level of statutory sick pay often their only entitlement, with relativity few employers offering more generous company sick pay policies, a situation exposed during the pandemic (Hayes et al., 2020). Unsurprisingly, considering these factors, recent research has highlighted the real risks of poverty faced by residential care workers and their families (Allen et al., 2022). The pay issues are exacerbated by ASC worker's contractual situations: zero-hours contracts with no guaranteed minimum number of hours are more prevalent than in other low-paid sectors (Migration Advisory Committee, 2022, p. 46–47). These working conditions act as cause-and-effect of ongoing national-level struggles with recruitment, retention, and turnover in the sector. There are currently 131,000 vacancies in ASC, and the turnover rate for direct care workers stands at 26.5% (Skills for Care, 2024a, p. 48). However, despite struggles to retain younger workers (Skills for Care, 2024a, p. 145–147), there is a core of long-serving workers (Skills for Care, 2024a, p. 65–67).

In response to the labor shortage and the end of intra-European Freedom of Movement post-Brexit, the government included paid ASC workers in its Health and Social Care visa scheme from February 2022 onwards. Skills for Care (2024a, p. 13) estimates 185,000 workers arriving in the UK between March 2022 and March 2024 commenced ASC roles. However, alongside this development, there has been a continuation of, and aggravation of, serious problems with the treatment of migrant care workers, which independent unions, such as United Voices of the World (UVW), treat as central concerns (Weghmann, 2023). Numerous examples of exploitation have occurred (University of Nottingham Rights Lab, 2022) and calls to a modern slavery helpline by care workers have increased dramatically (Unseen UK, 2023). In March 2024, the then Conservative government initiated a policy that meant migrant care workers would no longer be able to bring dependants to the UK. This move was strongly criticized as inhumane and redolent of colonial-era policy (Kenway, 2023), and fits within a broader picture of extraction of workers from poorer countries (Wichterich, 2020). Regarding numbers, indications are that this move has had an immediate impact: between April and June 2024 there were 81% fewer applications for the visa than in the corresponding 2023 months (Skills for Care, 2024a, p. 13). Such reactive changes to migrants' roles in paid care work (Kilkey, 2023) are indicative of short-term, politicized policy making.

### 1.3 Policy and care worker responses

There has long been a recognition that social care funding and provision, including the circumstances of workers, requires more radical structural change (Humphries, 2022). In his maiden speech as Prime Minister in 2019, Boris Johnson made a now infamous statement that his government would “fix the crisis in social care once and for all with a clear plan we have prepared to give every older person the dignity and security they deserve” (Hudson, 2021, p. 7). Now no longer in office, Johnson's statement has proved to be in keeping with recent talk of reform and lack of tangible action (Needham and Hall, 2023). Rather, recent years have seen a range of short-term measures, including on funding, and “a series of attempted reforms have been abandoned or delayed” (Needham and Hall, 2023, p. 288).

The DHSC has responsibility for ASC workforce strategy and development. The National Audit Office (NAO) delivered a critical verdict on the DHSC's ASC workforce reforms, describing progress as “slow” and noting delays to initiatives on training and workforce development (National Audit Office, 2023, p. 40). The mismatch in funds available for ASC workforce development when compared with the NHS is of note (Dromey and Hochlaf, 2018, p. 26), and the lack of a binding, resourced long-term strategy for the ASC workforce has been lamented (Foster, 2024, p. 36–37). This latter point has been addressed to an extent, with Skills for Care (a charity focusing on ASC workforce development and data) recently developing a strategy. However, the strategy, published in July 2024, has no implementation or enforcement behind it as things stand (Skills for Care, 2024b).

In 2022, the Scottish Government brought a National Care Service bill before the devolved parliament, but this has stalled and is facing increasing stakeholder opposition (Council of Scottish Local Authorities, 2024). The newly elected (July 2024) Labour government at UK level had the creation of a National Care Service in England in its manifesto (Labour Party, 2024, p. 100–101). Key to the new government's plans are improvements to ASC worker pay and conditions through sectoral-level fair pay agreements (Labour Party, 2024, p. 100), although implementation will take time. Paid care workers in England have lagged compared to counterparts in Scotland and Wales, where there is a Real Living Wage (RLW) guarantee, and where workers received a bonus payment in recognition of their contribution during the COVID-19 pandemic. In England since the new government was elected, a number of industrial disputes over pay in other public services have been settled (Elliot, 2024), and ASC workers could be forgiven for rueing the further wait for meaningful improvements to their situation. The extent of government influence on policy mentioned thus far—outsourcing of care provision and the sector's overall legislative and regulatory structures, control of migration, and workforce strategy—highlights its unique, overriding power to shape the quality and nature of paid ASC work (Hayes, 2017).

This final passage preceding the methods section considers what workers and those representing their interests do in response to their work circumstances. The extant literature identifies a variety of issues organizing revolves around in the ASC work context, many of which have been exacerbated by the direction of policy over recent decades. Furthermore, the valorisation of the individual in neoliberal capitalism has come at the expense of collectively oriented organizations like trade unions, for whom the turn to neoliberalism has had profound effects (Però, 2020, p. 902). This has been through specific legislation and action to decrease the power, influence, and capabilities of unions (Simms, 2024, p. 1308–1309), as well as the broader, interlinked cultural ascent of individualism, including the new work insecurities Sennett describes.

ASC workers' challenges are exacerbated by their lack of representation and influence and the fragmented nature of the workforce. Only 15% of private sector-employed ASC workers are unionized (Cominetti, 2023, p. 5), in contrast with 21% of all employees in England (Department for Business and Trade, 2023, p. 21). ASC workers currently lack any form of sectoral- or national-level bargaining (although it looks to be on the way), and neither do they have a recognized professional body to represent their interests. The benefits of greater professionalization of the ASC workforce, in part to address issues of low status and undervaluation, have been noted (Hayes et al., 2019; Hemmings et al., 2022), and this has been a cause for organizing in the sector (Weghmann, 2023). Returning to comparison with other devolved nations within the UK, unlike Scotland, Wales and Northern Ireland, ASC workers in England are not required to register (Hayes et al., 2019). Registration is regarded as an important component of professionalization.

Pay in ASC is among the worst in the labor market and, unsurprisingly it represents an important focus for organizing. Campaigning on various pay issues (Johnson et al., 2021) reflects the widespread dissatisfaction with renumeration. This includes basic pay, sick pay, pay for travel time between care episodes, and pay for breaks (Johnson et al., 2021; Nelson, 2019, p. 131; Smith, 2021). Equal pay has been a focal point in recent years, on gender lines, with local authority-employed workers in social care and other feminized sectors challenging their pay compared to better-paid work where men predominate (Murray, 2023). Returning to sick pay, that it has become such a focal point for organizing in this sector is emblematic of the working conditions. Sick pay's inadequacy is telling in work that is risky on numerous fronts: staff are regularly exposed to infection (Nyashanu et al., 2022), they are at risk of injury due to physical demands (Stacey, 2005) and potential violence from supported people (Kelly, 2017), and they are vulnerable to mental health problems due to the stressful nature of working environments and conditions (Nyashanu et al., 2022; Ravalier et al., 2019). That established unions, for example, have focused on it so much is indicative of how rudimentary the organizing demands are in this setting, and how poorly paid care workers are rewarded.

In addition to pay and conditions, there are contestations over worker status in this context: Anderson's (2010) study centers on Filipino migrant domestic workers' campaign to be recognized as workers for immigration purposes. These workers, and other paid care workers who are employed to work in supported people's own homes, face vulnerability through the blurring of work and non-work boundaries (which are complicated by familial, naturalized, and feminized, connotations). When such work is linked to immigration and citizenship status, the risks, including of deportation, are heightened. For instance, Alberti et al. (2013) describe the role of UNISON in campaigning for a group of Filipino migrant care workers—who were at risk of deportation due to new skills' requirements—to remain in the UK. As previously noted, there is a limited literature which focuses on paid care worker accounts, with their views on organizing often little understood (Whitfield, 2022). The next sections move on to explore these points empirically.

## 2 Methodology and methods

The data presented in this article is taken from 35 semi-structured interviews with key actors (organizers, administrators, founders) and paid direct care workers involved in organizing in the context of ASC in England. The organizations participants worked for or were members of included both independent and established trade unions. Independent unions arose in the 2010s largely due to differences and dissatisfaction with established unions and included groups of workers—overwhelmingly of migrant backgrounds—breaking away to form and/or join other workers in independent unions (see Però, 2020, p. 905–907; Weghmann, 2022, p. 136).

Table 1 provides additional detail in relation to the key actors interviewed including their pseudonym (given for all participants), the name and type of their organization (pseudonyms were used for organization names where permission to use original name was not granted). In order to preserve key actor anonymity, specific role titles for individuals have not been provided, and their demographic information was not sought. Table 2 provides details on which organizations paid care workers were members of or involved with, alongside their demographic characteristics. Of the 16 paid care workers interviewed, five were men and 11 women. The average age of the workers was 47, which is close to the ASC average of 44 (Skills for Care, 2024a, p. 76).

**Table 1:** Key actor participants.

**Key actor participant (pseudonyms)**	**Organization name**	**Type of organization**
Sarah	United Voices of the World (UVW)	Independent union
Silvia	UVW	Independent union
Paul	WorkTogether (pseudonym)	Established union
David	WorkTogether (pseudonym)	Established union
Louise	WorkTogether (pseudonym)	Established union
Amanda	WorkTogether (pseudonym)	Established union
Ruth	Care and Support Workers Organize (CaSWO)	Campaign group
Wendy	CaSWO	Campaign group
Livia	CaSWO	Campaign group
Catherine	CaSWO	Campaign group
Lorraine	PeoplePower (pseudonym)	Community organizing group
Steve	PeoplePower (pseudonym)	Community organizing group
Lorrie	Homecare Workers' Group (HCWG)	Peer support and campaign group
Kathryn	CollectiveWorkers (pseudonym)	Established union
Lesley	CollectiveWorkers (pseudonym)	Established union
Amala	CollectiveWorkers (pseudonym)	Established union

**Table 2:** Paid care worker participants.

**Worker participant (pseudonyms)**	**Organization name and type**	**Gender**	**Age**	**Ethnicity (self-defined)**
Rita	CollectiveWorkers (established union—pseudonym)	F	47	British Indian
Tracey	PeoplePower (community organizing group—pseudonym)	F	28	British
Kim	WorkTogether (established union—pseudonym)	F	56	White British
Judith	WorkTogether (established union—pseudonym)	F	36	White British
Sonia	WorkTogether (established union—pseudonym)	F	36	White (non-British)
Claire	WorkTogether (established union—pseudonym)	F	45	White Northern Irish
Laura	WorkTogether (established union—pseudonym)	F	48	White British
Carla	RepresentStaff (established union—pseudonym)	F	56	White Mixed
Elena	UVW (independent union)	F	70	White (Spanish)
Cynthia	UVW (independent union)	F	48	Latin American
Florence	UVW (independent union)	F	39	Black Caribbean
Tom	WorkTogether (established union—pseudonym)	M	65	White British
Clive	WorkTogether (established union—pseudonym)	M	33	White British
Bruce	WorkTogether (established union—pseudonym)	M	53	White British
Claude	UVW (independent union)	M	45	Black African
Ade	UVW (independent union)	M	54	Black African

The semi-structured interviews were conducted in-person (including at UVW's office, and at the author's university), online (via Zoom), and by telephone. Interviews took place between December 2023 and July 2024 and lasted approximately an hour on average. Key actors and paid care workers were recruited for different purposes: the former to gain organizational perspectives, and the latter for insights on workers' micro-level experiences. The interview schedules included questions on the general picture of organizing activity, barriers and successes in relation to it, and the care worker interviews additionally examined worker motivations around involvement in organizing. Both types of interviews focused on exploring key areas of focus for ASC organizing, the main priority of this article.

Participants were selected due to their involvement in organizing in this context, their role within respective organizations, and their status as direct care workers. This sampling was therefore targeted and purposive (Clark et al., 2021), and reliant on a variety of recruitment strategies. The UVW and WorkTogether samples were recruited through academic colleagues with existing connections to people in the unions. Other participants, such as those in the campaign group, CaSWO, were recruited by snowballing once the author had made initial contact with the organization.

The interviews were recorded and transcribed in preparation for analysis. In accordance with the ethical approval gained through the university's formal ethics procedures individual participants were given pseudonyms, and other identifying features, such as specific job titles or places of work, have been changed or omitted. Where organizations are named, they provided explicit permission, whereas in other cases permission was given conditionally and pseudonyms are employed.

The analysis for this article was thematic in line with the method of Braun and Clarke (2006). The results are presented in headings of key themes identified through comprehensive coding of the data and constitute summarized groupings of the issues and causes their campaigning and activism centers on. Due to the sample size and strategy, theoretical saturation could not be assumed. The study is illustrative (of the analytical themes) as opposed to extensive, with quotes utilized to exhibit the range within themes (rather than a comprehensive picture). However, the strength of the approach is in “developing a much richer understanding of processes, motivations, beliefs and attitudes than can be gained from quantitative research” (Rowlingson, 2002, p. 632).

## 3 Results

This section presents findings under four headings, *Pay and conditions, Systemic/structural change, Awareness-raising and being heard*, and *Environment and practices*. These headings were identified through examination of key actor and ASC worker accounts and include factors participants highlighted as being sources or concern or areas they wanted to see change (but were not necessarily the focus of related campaigning or activism) regarding their work situation. It should be added here that the organizations involved are varied, and as such, the nature of their work and aims differs in certain regards, such as their organizing strategies. While this variation was useful to ensuring a range of organizing was captured, this analysis brings together, and emphasizes, the collective breadth of issues these varied actors seek change on. Variation in terms of organizing scope and strategy, including comparing between independent and established unions, is given attention elsewhere (see Però, 2020).

### 3.1 Pay and conditions

Much of the focus of the campaigns of paid care workers and those representing their interests is on the persistently poor pay and conditions. Participant accounts chime with what the literature says about the wider picture of work and pay in ASC (Hayes, 2017; Foster, 2024). For CaSWO, paying care workers better was fundamental to the organization's foundation and call to action:

I gave 15 years to this sector, and I can't get them back, so they need to pay now … it was our lives, and we are genuinely a group of women who see the pressures that are on women. Then we came home from work and then we realized that we couldn't go on nice holidays with our mates, or we couldn't afford to get a car, so we were always on buses. That's hard, being poor when you really are working really, really hard. So, it was our lives. (Ruth, CaSWO key actor)

This quote is fundamentally about low pay, and about it being a gendered issue in the care work context (England, 2005), and it touches on workers' experiences of poverty (Allen et al., 2022) and exclusion (Gil, 2022). Paid care workers' circumstances gained enhanced visibility during the pandemic, in part through the appreciation shown with the Clap for Carers (Manthorpe et al., 2022) gesture. In the UK, as in other European countries, on Thursday evenings during the pandemic, this was the public's clapping for carers, on their doorsteps or balconies, to thank them for their efforts. However, despite this short-term recognition and gratitude, it also provoked frustration among care workers. During this time Cynthia, a UVW (independent union) member and care worker, was asked, by the manager of the care home where she was employed, to acknowledge people's clapping:

The manager said that we must go to the window because people are coming on the street to clapping and with cards saying, ‘thank you'. And said, ‘Cynthia, go'. I said, ‘No, I'm not going to go, I don't want claps, I just want a better salary'. And the manager… my colleagues told the manager, and the manager was upset. And I said, why are you upset, I don't want to go do that, I just want better pay. Pay more and that's it, I don't need claps. (Cynthia, UVW care worker)

Cynthia's frustration and anger was provoked by the perceived tokenism of Clap for Carers, but also by the treatment she and her colleagues had from their employer, particularly during the pandemic. The increased risks they faced then were not factored into how they were rewarded, and the workers did not feel enough was being done to mitigate the dangers the of working through the pandemic (Nyashanu et al., 2022). Since the pandemic, there has been a rise in inflation and living costs, yet care worker pay has not kept up leaving some workers struggling to meet basic costs (Care Quality Commission, 2023, p. 98–99). Another point made about levels of pay was the disparity with NHS workers and their superior pay entitlements.

The community organizing organization, PeoplePower, have hourly rates of pay as one of their core causes: they clearly recognize the importance of basic pay, but their work is not limited to this issue. They have campaigned on pensions for low-paid workers, and on trying to improve contractual situations for workers whose contracts do not guarantee a certain number of hours, or whose hours are unpredictable or changed at short notice. In addition to these deficits, campaigns also draw attention to instances where care workers have no option to pay work-related costs out of their own pocket. CaSWO (a care worker campaign group) campaigned on the issue of parking charges for homecare workers in London, for example, in attempts to build that into local authority commissioning and not leave workers to shoulder the cost.

Beyond basic pay, there were several other dimensions of pay discussed in interviews, which amount to limitations in paid care workers' occupational welfare. These included sick pay. These challenges have been about the inadequacy of statutory sick pay, and conditions shaping sick pay payment, such as it not becoming available until workers have been off for 3 days. Established unions have fought big campaigns on this, including with large care providers, or in particular regions of the country (Hayes, 2020). These were given urgency and impetus by the pandemic when workers faced situations of not wanting to spread the virus when unwell, but also not being paid if they did not work (Hayes et al., 2020). Away from the pandemic context, paid care work ordinarily carries several risks, including for example, of musculoskeletal injury due to its physical demands (Mabry et al., 2018). These risks are compounded by inadequate financial support (sick pay) to manage these injuries or take time off when they happen:

Sometimes I saw the staff…complaining, working, I don't know, ten years in that place, and they are in pain in their knees, they need a knee replacement, things like that. But they have to use their holiday to use for absence, for sick absence. And I said that is not right, if you are sick, why are you here? (Cynthia, UVW care worker)

These indignities are part of wider degradations of working conditions, with inadequate pay for work-necessary travel time, for sleep-in shifts, for breaks, for training, or incremental pay tied to length of service among the other issues raised. Returning to Ruth's opening quote of this sub-section, these concerns feed into a wider picture of care worker risk of poverty. The sense from care workers and those who represent them is of heightened injustice that they face these situations when they work so hard and contribute so much:

It is completely wrong that people who were doing some of the hardest work and really important work in terms of holding our communities and societies together and taking care of loved ones, they're not recognized for it, they're not rewarded for it and often they're treated quite badly for it. (Sarah, UVW key actor)

### 3.2 Systemic/structural change

Although much of what paid care workers and those representing their interests campaign on and want to change relates to pay and conditions, there was awareness that these issues are situated within a system characterized by significant broader structural problems. The overall message coming from participants was bleak, and at times this extended to their views regarding the prospects for change. One care worker, Judith, a WorkTogether (established union) member, was blunt about the state of ASC:

It's a broken system, it's a massively broken system. I mean, I could go on all day about it. (Judith, WorkTogether care worker)

These views mirror much political rhetoric about a system that needs “fixing” (Hudson, 2021), despite evidence of a lack of tangible action (Needham and Hall, 2023) toward that end.

In addition to highlighting some of the things that are wrong, or do not work, participants themselves raised potential solutions. WorkTogether has campaigned for the introduction of a National Care Service in England, with key elements of this returning ASC services to public delivery, and working toward ASC being on a more equal footing to the NHS:

I think fundamentally it's about that transformational shift within the sector around the model of delivery. And I think that also links into obviously the union's national campaign around the National Care Service that has sort of parity with the NHS, publicly delivered. (Paul, WorkTogether key actor)

It is important to note that public delivery runs counter to the direction of government policy in recent decades, which has focused on privatization and market-led service provision (Horton, 2022). Aligned with this pursuit of service insourcing, WorkTogether has sought to challenge current commissioning practices. Part of their approach has been to strategically focus on the role of local authorities as key power holders within ASC:

Our strategy was moving from an employer focused strategy to a commissioner focused strategy, so instead of focusing on employers as decision makers, it was the councils as key influencers, because they obviously have responsibility for commissioning social care, and they have within their control minimum commissioning standards and stuff like that. (Paul, WorkTogether key actor)

One of the things that a National Care Service may bring about would be increased funding for ASC, which alongside factors like public delivery and cultural change, was mentioned as an area where reform was imperative:

I think the key is to secure a commitment for increased funding from central government down to local authorities, who can then hopefully spend more on social care, and that unlocks so much in terms of the potential for improving employment conditions for care workers. [Lorrie, HCWG (peer support and campaign group) key actor]

Clear features of the ASC workforce include its dispersal and fragmentation, and unevenness, and these are aspects that system-wide improvements could potentially alleviate. System-wide change could be more narrowly directed toward workers, through increased regulation, standardization or moves toward professionalization (Hemmings et al., 2022). As part of this, introducing paid care worker registration in England—it is a requirement in Scotland, Wales, and Northern Ireland (Hayes et al., 2019)—is something unions have campaigned for:

The other thing [CollectiveWorkers]' been campaigning for a while is in England, there's no sort of carer registration … we think it would professionalize the industry and give the care workers a higher status more on par with nurses because nurses have to be registered. (Lesley, CollectiveWorkers key actor)

Lesley points out that a potential consequence of registration would be to enhance the status and value of paid care workers, which would be in contrast with their generally poor treatment overall (Lynch, 2022).

Tronto's research (2017) highlights the exploitation of care workers, including across borders. Migrants are key constituents within the ASC workforce and have in recent years been central to UK government attempts to shape numbers in the care workforce (Foster, 2024, p. 44–52). Their role has been politicized, and participants highlighted injustices stemming from Health and Social Care visa rules, including around employer sponsorship and new conditions preventing workers from bringing dependants with them to the UK:

It didn't take too long for us to come to the conclusion that this work is badly paid because we are women, and it didn't take us very long to say, ‘Well, do you what? They're denying women the right to dependents coming to this country, because it's an extension of colonialism'. (Ruth, CaSWO key actor)

The one that we're looking to develop an approach to is issues specific to migrant workers and the exploitation of migrant workers by providers. The use of those visas to really exercise control over these people, I think that's one that desperately needs cracking, and we need to develop a strategy really fast. (David, WorkTogether key actor)

The work of UVW in ASC has centered on the cause of migrant care workers, in line with the union's core principles of supporting migrant and minority ethnic, low-paid workers [United Voices of the World (UVW), 2024].

### 3.3 Awareness-raising and being heard

A key part of what these organizations and workers do is raise awareness of ASC, and of paid care work and what it involves. This is intimately tied to the issue of representation, as the reason these organizations see the need to raise awareness is that care workers lack adequate representation or avenues where their individual and collective voices have a say in or inform decision-making (Hayes et al., 2019). This lack of representation is also linked to greater fragmentation, which stems in part from ASC's marketisation (Corlet Walker et al., 2022). One of the important points to come from the accounts, particularly from the care workers, is that they are not listened to by various actors in a range of contexts. Therefore, efforts to raise awareness involve pushing back against this, and competing with other interests in areas such as policy or trade union prioritization.

A fundamental part of CaSWO's original and ongoing purpose has been to raise awareness of the current situation in ASC:

To highlight the absolute broken mess which is social care. Also unpicking the power dynamics in social care … when we spoke at the start, it was really important that we stood alongside disabled people, and people who use services, to highlight just how bad care services are, so both looking at workers' terms and conditions but also a failing system. (Ruth, CaSWO key actor)

There are three connected issues here that CaSWO have communicated in their work. Firstly, they recognize the shared interests between care workers and disabled people, and secondly, relatedly, between job quality and care quality (Burns et al., 2016). Thirdly, linked to the previous theme, they acknowledge that the problems faced by workers and those who draw on services are part of a bigger picture of a sector or system that has multiple, systemic, under-addressed failings.

A major frustration for care workers is that they are not listened to by their employers, and this has been a motivating factor for some in joining a union. For example, Kim joined WorkTogether during the COVID-19 pandemic because she had an underlying health issue that increased the risks of this frontline work:

I said to them I shouldn't be working, and this is why I decided to join a union, because I just felt they were not listening, they were not listening to me. (Kim, WorkTogether care worker)

Although central to unions' remit is to represent workers, others saw raising awareness of ASC with unions as central to their aims. For CaSWO, an organization where members are involved with or even employed by unions, getting care workers' circumstances on union agendas was another aim:

What CaSWO is about is also about making sure that … pushing this stuff, yeah, within our trade unions, to make sure that it is something that they are paying attention to and giving time and resources to and stuff like that, as well. (Wendy, CaSWO key actor)

Although these findings highlight concerns about employers and unions not listening to care workers, there was a frustration among some workers at a more general invisibility at societal level wherein care workers voices are not heard:

We give everything for this job, but people don't see us. They don't listen to us. They don't hear you. (Claude, UVW care worker)

The invisibility felt by care workers is connected to, and makes more challenging, the efforts that they make to highlight their work's importance and complexity. The former involves countering the lack of awareness of what paid care workers do, and challenging perceptions:

The way that society sees care work, you know, they look at it as something that not many people would want to do. They say, ‘that's disgusting', it's not valued. I don't feel it's valued at all, even though like during the pandemic it was more, maybe for that short period, you know, you're a care worker blah blah blah, but then it's been forgotten again. (Sonia, WorkTogether care worker)

Even although the pandemic provided a short-term increase in public cognisance of paid care work, as Sonia points out, this has not been maintained or capitalized upon. Sonia's quote points to the stigma of care work (Ashforth and Kreiner, 1999), with its connotations of dirty work (Stacey, 2005), which Florence too noted:

I find that we struggle because okay, when you go out and you say to somebody, ‘I'm Florence, I am a professional carer', it's like everybody thinks that oh yeah, she's just a carer. They don't see that the job that I do on a day-to-day basis is something that needs to be recognized … carers don't really get that recognition, it's like we're not part of the workforce, the society, it's like we've been pushed behind… we've been pushed to one side. We are just carers that clean the bum. People don't recognize that a carer is more than that. (Florence, UVW care worker)

Not only is this aspect of personal care stigmatized, but paid care workers argue that their work is essentialised around it, and that when people do see this work, this is all they see, or cannot see past. Florence's observation that she is seen as *just a carer* is particularly revealing as it undervalues the contributions care workers make and symbolizes paid care work's low status. The quote also hints at the exclusions of paid care work (Gil, 2022), revealed through Florence's point about being “pushed behind” or “to one side,” and which manifest themself further through very low pay and inadequate working conditions.

This challenging of perceptions is about paid care work receiving the recognition that workers, and those representing their interests, feel it deserves. This was key to the establishment of HCWG:

I'm really, really keen that Homecare Workers' Group might help do something to change perceptions of care work, where people do have perceptions of it being dirty work, unskilled, women's work, relating to working-class people, and a lot of negative stuff that goes along with that, and actually show that it is skilled, it is medicalised, it involves a high level of responsibility, and it has social value that is off the charts. And I tend to think that there's already got to be an understanding of that, based on the Clap for Carers in COVID. (Lorrie, HCWG key actor)

Lorrie's quote points to two-pronged work of challenging negative perceptions and promoting positive aspects that this complex, important work generates. Of note is her mention of Clap for Carers as stimulating interest and awareness of paid care work, hinting at it being a reference point or something to build further awareness-raising upon.

Much of the awareness-raising discussed has been broadly aimed at the general public or societal-level perceptions, but participants specifically spoke of the need to educate young people about care and care work:

I actually think they start in schools …. This is education in schools and recognizing when you go into careers that social care is an avenue to look at…I don't think schools and colleges do enough to make that as exciting as nursing, as becoming a doctor, as other health professionals and I think that's where we fall down. And that comes with, again, a societal change with how we recognize the workforce. (Kathryn, CollectiveWorkers key actor)

### 3.4 Environment and practices

Participants identified several elements of the environment and practices of paid care work that were problematic and where they want things to change. One factor that deems paid care workers' low levels of unionization or wider occupational representation surprising is the levels of risk involved in the work, particularly around health and safety. These risks are bound up in a range of other systemic problems, in particular ongoing workforce issues with recruitment, retention, and high turnover (Dromey and Hochlaf, 2018).

The risks apparent in care work settings are varied, but one issue was mentioned by several participants as being a fundamental driver, that of chronic understaffing. One of the WorkTogether members, Claire, who had returned to ASC recently after a few years working in children's social care, noted the decline in this regard:

I'm feeling really sad at how things have taken a bit of a turn for the worst with care work, like there's a lot of shortages of staff, not the right staff being employed, you know, holes in training ... There just seems to be a lot of gaps since I … and I don't know if it's really Brexit when lots of people moved away and they struggle to employ people now. And, obviously, COVID's had an impact, as well. But I see a big change in adult care since I've come back, you know, they're just permanently short-staffed. (Claire, WorkTogether care worker)

It is interesting to note the decline felt by the worker, and the sectoral-level figures of recent years show a continuing inability to resolve the labor shortage. In 2019, there were 122,000 (Skills for Care, 2019, p. 59) vacancies in ASC with that figure remaining high, and at 131,000 currently (Skills for Care, 2024a, p. 48). Not only does Claire lament the insufficient numbers, but she also questions the suitability of some of the workers who are taken on. “Holes in training” was highlighted by others as problematic in terms of the responsibilities involved. This creates risks for the care worker potentially making errors, and for the supported person if they receive the wrong medication or if it is not administered correctly:

They cannot expect somebody to do medication training online, be signed off by somebody who is not competent themselves and then be expected to go and administer medication, controlled medication, all these things and then wonder why there are errors. I think the responsibility of the support worker is unbelievable. (Judith, WorkTogether care worker)

Understaffing feeds other problems as it places workers in compromising situations: one worker said she was no longer prepared to work in care homes because of the staffing levels and their implications:

You're always understaffed. There's too much to do. You're doing one-on-one instead of two-on-one with people, which is dangerous for them and hurting yourself. (Laura, WorkTogether care worker)

Understaffing intensifies risk for care workers who may have little choice but to provide personal care support, for example, on their own when two people should be doing the work. As Laura highlights, this also increases risk for the supported person and thus potentially impinges on care quality. This concern for care quality and standards of the support given is at the heart of much of what paid care workers want to see change in their work situation. A key actor from WorkTogether, David, described this as “a huge issue for our membership,” which “comes up with the vast majority of our members.” David connected this to care workers' commitment and attachment (Ravalier et al., 2019) to the work that they do:

So, say if you're working in a care home and you're on a ward with like dementia patients and there's 12 residents there and you're on your own, it's a horrible position to be in, but also … so there's that individual aspect to it. But also, these people are in this sector for a reason, especially the experienced staff that have been 20–30 years, it's a vocation, because otherwise why would you be doing it? (David, WorkTogether key actor)

The practices of paid care work, or the labor process, are intrinsically relational, and as such, changes in the situation of workers are likely to have considerable impact on those drawing on support. Paid care workers tend to have strong work attachments (Stacey, 2005), especially to the people they support (Daly, 2023). This can influence choices regarding potential participation in organizing (Boris and Klein, 2006; Duffy, 2010), particularly strike action (Cranford et al., 2018; Whitfield, 2022). This does complicate the motivations of workers around organizing and campaigning, with some reticent due to fears gains for workers might come at the expense of care services (Mareschal and Ciorici, 2021, p. 349) or resources, while others more boldly espousing that good quality of care relies on staff being treated well (or better than they currently are). One of the slogans used by UVW in their campaign to improve working conditions at a London care home was: “Quality care deserves quality pay.”

Returning to health and safety, the pandemic was key to unions and other organizations representing workers adapting their responses to health and safety issues (Martínez Lucio, 2020). The pandemic highlighted the connections between health and safety and wider employment conditions, particularly with the problems over Personal Protective Equipment (PPE) supply and the inadequacies of sick pay. CaSWO started during the pandemic with these issues as core causes, but Ruth argued, the wider connections were clear:

It was absolutely health and safety, but it doesn't take long to realize how interlinked health and safety, and terms and conditions are, right? As soon as when people were getting sick and they couldn't go into work, or they'd got the virus and they couldn't go in, and then they weren't getting paid. (Ruth, CaSWO key actor)

Other issues highlighted included the risk of injury—again related to short-staffing—resulting from the potential aggression of supported people, which poor quality service provision can exacerbate:

If they increase the ratios of staff to residents like a lot of these attacks [on staff] … I think they'd happen less frequently. And I think if something did happen, there would be people on hand to help straight away. (Lesley, CollectiveWorkers key actor)

Beyond the immediate risks for supported people and care workers' role boundaries, such scenarios have implications in relation to issues with particular salience in ASC. These include skills development and progression opportunities, which themselves are key to the broader question of ASC worker professionalization.

## 4 Discussion

The range and number of issues raised by participants draws attention to the extent of the problems they face in the ASC work context. This section considers what the identification of the issues says about the standing and quality of paid care work, and the relative status of the workers. It then discusses what this suggests about the potential for improvements, including the present and future role of organizing in driving those. The section concludes by returning to the theoretical literature to aid interpretation of these accounts, and to posit that the actions and concerns they describe represent a care-centered opposition to neoliberal capitalism.

The first thing to observe about the first theme, pay and conditions, is that the causes are basic, at the level of rudimentary employment rights, and are indicative of a lack of care toward these workers. Ruth's quote about doing the work for 15 years and still being poor and socially and economically excluded succinctly articulates the endemic, normalized, and long-standing character of these employment degradations. Furthermore, these are deep-rooted and extend beyond basic pay to a suite of other pay indicators. That sick pay has been so central to campaigning in this sector is emblematic of the indecency of conditions, although there are signs of this being belatedly addressed through the new UK government's Employment Rights Bill (UNISON, 2024). The basic nature of many of these demands also indicates that there is far to travel to the achievement of meaningful change when the starting point is so low.

Participants did not view pay and conditions in a vacuum, but as part of broader systemic/structural change to ASC, the second grouping identified. Their concerns revealed a wider awareness of this employment's location in a social care system beset by problems and inadequacies (Hudson, 2021; Humphries, 2022). The range of problems and potential solutions outlined attests to the extent of difficulties facing the sector, including the workers. Crucially workers associated their micro-level experiences of pay and conditions with wider processes through their association with negative impacts on care provision and care quality for supported people. Their accounts represented a resistance or opposition to the neoliberalisation of care work, with outsourcing (Però, 2020) perceived to be a particularly negative dimension. Furthermore, those involved in organizing were strongly opposed to more general aspects of the organization and place of care within contemporary capitalism, including its marked over-reliance on women. Workers and their representatives recognize paid care work's embeddedness within, and role as driver of, social inequalities. Negative comparisons were drawn with the NHS, including over worker representation and work status, as nurses employed in the NHS—as with those in other national contexts (Stanton et al., 2022) – have stronger collective power through union coverage and representation. This inequality compounded the sense of ASC's lowly place within employment hierarchies, and participants' desire for greater professionalization of the ASC workforce (Hayes et al., 2019) sought to counter that. The general treatment, which at times results in exploitation of migrant workers, too was the source for much lamentation. The overall sense was of a series of small and larger complaints about immediate workplace issues playing out against a backdrop of systemic failings and societal and political-economic priorities that participants were strongly resistant to.

Raising awareness of paid care work's importance and complexity, and pursuing ways for paid care workers to be heard and to influence, was the third theme. That participants felt these steps to be necessary are in themselves indicative of neglect and sidelining of care workers, and in keeping with the broader picture of poor pay and conditions and systemic/structural failings (Humphries, 2022). Paid care workers were riled by their employers not listening to them, and this fitted within workers' broader perceptions that they were not seen or listened to at societal level. The experience of not being listened to communicates that one's views are not important, and this is damaging for workers whose status is undermined in many other ways. CaSWO saw an important part of their role as raising awareness of paid care work issues within unions, which points to ASC's struggles for prioritization in multi-sector unions and its relatively weak policy traction. That said, the data contained evidence of considerable sustained, prominent campaigning on ASC in established unions at both national and regional levels. The points raised by Kathryn about education represent more long-term strategies to raise awareness of care and care work among future generations. That education is deemed necessary also points to the ingrained nature of views about the position of care work. It also indicates the scale of the issue and substantiates participants' contention that collective solutions are required to challenge current everyday understandings.

The issues participants campaign on regarding environment and practices, the fourth theme identified, such as the risks accentuated by chronic understaffing, are closely and cyclically intertwined with the work's poor pay and conditions and lack of status. Other aspects of working environments, such as the shift patterns, have real impacts on worker's non-work lives. Other important implications of these findings are of the weakness of various elements of regulation in ASC, which relate closely to worker orientations toward organizing. Dangerously low levels of staffing, which bring risks to supported people and workers (Kelly, 2017; Mabry et al., 2018), are not sufficiently addressed, and they influence and are shaped by turnover problems. Workers raised concerns about the systems in place to screen workers for suitability, which is again inseparable from providers' urgent need for staff. The issues over staffing levels heighten the responsibilities for workers in posts, and this has an important bearing on decisions around organizing, primarily through concerns that workers' actions might come at the expense of the people they care for.

These four groups of issues clearly show that paid ASC work is characterized by various degradations, and that there are several ways its status is undermined and rendered as low in occupational hierarchies. The data presented provides a comprehensive view of the breadth of concerns held by paid care workers and those who represent their interests and enhances understanding of their needs and demands. Although there are signs that some change may be coming with the new government's plans (Labour Party, 2024), the basic nature of the issues organized around indicate that there is a long way to go on multiple fronts, before this work catches up with conditions taken for granted by other workers, such as those in the NHS. With regards pay, reform ought to be wide-ranging and deep-rooted across the numerous indicators where ASC workers are so poorly compensated.

On the prospects for organizing, there was a sense of exclusion felt by workers, with Florence's comment that people see her as “just a carer” telling. There was a tangible sense of weariness from some participants that challenging perceptions was a losing battle, but at other times, there was push back. In this way, participants advocating for the importance of care work is in keeping with the sense of care as constitutive of opposition and resistance, or an alternative, to the ontological basis and ideology of contemporary neoliberal capitalism (Tronto, 2017). It is apparent that those involved in organizing seek collective solutions, often in response to structural and economic failings in ASC, including those relating to its reliance on private provision (Hudson, 2021). Their views represent a rejection of the way care work exists and is imagined within neoliberal capitalism (Lynch, 2022), including through outsourcing (Pollock et al., 2005) and degradation of working conditions (Hayes, 2017). Although again it is worth emphasizing the urgent, immediate nature of many of participants' claims, workers did imagine care's current character and standing to be part of a broader struggle to challenge social inequalities. Learning from the work of Tronto (2017) and Lynch (2022), and others, enhances this article's scope and its development of a thorough and rounded take on workers' opposition to the way their work is currently, and its conception and place within wider societal structures.

ASC workers' lack of representation, as evidenced through low levels of unionization and there being no recognized, national professional body, is a factor in the continued poor quality of this work. There have been successes, though, including among the organizations we interviewed. For instance, UVW achieved a number of wins for workers at a nursing home in London, and the established unions have won regional or provider-specific gains on issues such as sick pay and living wages. Workers' fragmentation and dispersal are significant barriers to organizing, and access is further compromised by employer hostility to trade unions (Nelson, 2021). Furthermore, the complex structure of ASC delivery and funding makes it difficult to establish where blame or power lies, or which “sources of authority” to target with action (Boris and Klein, 2006, p. 90). This article's findings show that there are multiple targets of existing action include national government, local government, and care providers. In order for successes to be replicated, and for organizing to have broader influence, information about unions and organizing needs to reach more care workers, and unions require access to greater numbers of workplaces. This is likely to result in further opportunities to enhance the circumstances of ASC workers.

To conclude, this article improves understanding of the needs and demands of paid care workers, and what they want to see change, based on their own—rarely platformed—accounts and those of organizations representing their interests. They are not merely opposed to being poorly paid or having an insecure contract, but hold a broader dissatisfaction with how they are treated, and how care and care work stand and are imagined within contemporary neoliberal capitalism. This extends to care's role in perpetuating intersecting social inequalities. The findings show the breadth and depth of concerns at such anti-care tendencies, and the analysis converses with relevant care literature to bring out the care-centered elements of workers' opposition.

Although this article, building on contributions, including those of Johnson et al. (2021) and Whitfield (2022), strengthens the fledgling literature on organizing among paid ASC workers in England, there is much that is still unknown. There remains a lack of robust, sector-wide data on trade union membership, for example, including key characteristics such as member demographics and particular concentrations of membership among paid ASC workers. Better understanding of the diverse nature of pay inequalities among ASC workers is an area that warrants additional academic attention. Furthermore, monitoring the impact of planned reforms as they pertain to issues across the four identified findings themes is necessary moving forward.

## Data Availability

The original contributions presented in the study are included in the article/supplementary material, further inquiries can be directed to the corresponding author.
